# The Advances in the Special Microwave Effects of the Heterogeneous Catalytic Reactions

**DOI:** 10.3389/fchem.2020.00355

**Published:** 2020-05-05

**Authors:** Hong Li, Chunyu Zhang, Chuanrui Pang, Xingang Li, Xin Gao

**Affiliations:** ^1^School of Chemical Engineering and Technology, National Engineering Research Center of Distillation Technology, Collaborative Innovation Center of Chemical Science and Engineering (Tianjin), Tianjin University, Tianjin, China; ^2^TJU Binhai Industrial Research Institute Limited Company, Tianjin, China

**Keywords:** microwave, catalysis, special thermal effect, plasma, process intensification

## Abstract

In the present, microwave field has been widely used in chemical processes as an important means of intensification. The heterogeneous catalysts coupling with microwave has been shown to have many advantages, such as high catalytic performance and stability. Our objective is to focus an up-to-date overview concerning the advances in the special microwave effects of the heterogeneous catalytic reactions including special thermal effect, microwave plasma, enhanced active groups, and the flexibility of structure. This review systematically states the action mechanism and some practical application of microwave-induced catalytic process. Finally, the potential research directions in the field of microwave-induced catalysis are prospected.

## Introduction

As a common way of process intensification (PI) technology, microwave field, which is constituted of alternating magnetic and electric fields, has the advantages of volumetric heating, energy saving and higher selectivity (Chandrasekaran et al., 2012; Binner et al., 2013; Roy et al., 2017), and has been more and more popular in various chemical processes. In the courses of relative volatility, reactive distillation, drying and membrane separation (Li et al., 2017, 2019c; Gao et al., 2018), the addition of microwave irradiation can greatly facilitate the process. At the meantime, a great number of scholars have introduced microwave into the field of heterogeneous catalytic reactions (Gao et al., 2019; Li et al., 2019a,b; Zhang et al., 2019). Earlier microwave-assisted catalytic reactions can be traced back to 1992. Adámek and Hájek (1992) applied microwave to the reactions of tetrachloromethane and ethyl trichloroacetate with styrene in the presence of metal complexes. The reaction rate of microwave irradiation can be increased for several folds compared to that of conventional heating. As a result, the synergistic use of microwaves and catalysts emerged in multiple forms and reactions. In order to expand the practical application of microwave, it is necessary to study the mechanism of microwave action. As good microwave absorbers, solid catalysts can be heated rapidly due to high dielectric properties such as carbon particles. In terms of molecular level, polar molecular can rotate rapidly with the alternating electromagnetic field and produce ions migration (Sajjadi et al., 2014; Hirota et al., 2017). In the process of microwave irradiation, the molecular thermal motion increases and the heating efficiency is determined by molecular dipole (Oh et al., 2017). In addition, the structure of solid catalysts supported by polar functional groups or enzymes can change under microwave field for the special function.

In this paper, we state various effects of microwave on catalysts and classify according to the mechanism. Microwave can accelerate the reaction by the special thermal effect, plasma, special active effect and changing the structure of enzyme. This review analyzes and summarizes various influences through practical reactions. After introducing the microwave-induced process, we put forward some unsolved problems for the mechanism and the prospect of its application. The review can give the following scholars better guidance and understanding of microwave-induced heterogeneous catalytic technology.

## Special Thermal Effect of Microwave on Solid Catalysts

**Scheme 1:**
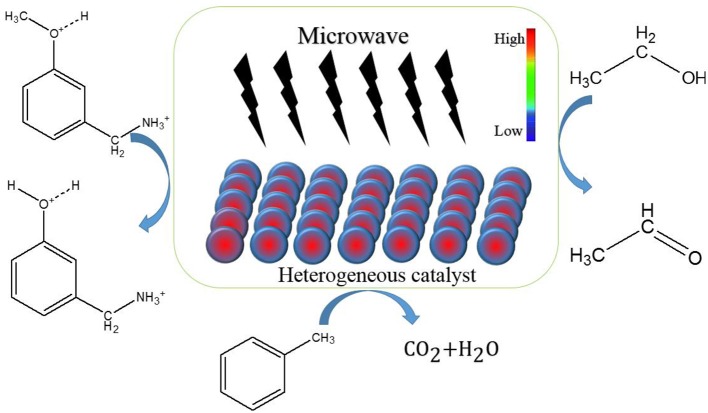
The schematic diagram of the special thermal effect of microwave on solid catalysts.

Thermal effect of microwave is the phenomenon that microwave energy is absorbed by dielectric materials and converted into heat energy, which is shown as the total loss of microwave energy in materials. The special thermal effect, as shown in Scheme 1, is also known as hot spot and has been discovered by many scholars (Jiang et al., 2018; Vakili et al., 2018).

For liquid phase reactions, the temperature of solid catalysts is higher than that of solvent owing to special thermal effect. Bogdal et al. (2002) found heterogeneous thermal effect by using Magtrieve which is an oxidant on the basis of tetravalent chromium dioxide (CrO_2_) that can absorb microwave and convert energy into heat. It was found that the temperature the center of solid catalyst is higher than other parts and solvent in the microwave field. The oxidation of alcohols to carbonyl with Magtrieve under microwave irradiation was following researched. The higher temperature of the solid support can lead to higher conversion of reactants and higher reaction rates. The special local thermal effect of microwave and Magtrieve will provide a good reaction environment to promote oxidation reaction. Owing to the high temperature of the solid catalyst in the microwave field, they got higher yield and save the reaction time in comparison with conventional heating. Mishra et al. (2019) degraded Brilliant Green (BG) by means of microwave-assisted catalytic spinel zinc ferrite (SZFO) sheets. SZFO firstly adsorbed BG and subsequently is exposed to MW irradiation under the output power of 360 W at 2.45 GHz. The result showed that 99% of the total removal efficiency can be obtained within 5 min and got inorganic ions. SZFO can efficient absorb MW energy, which form a local high temperature area at the catalyst surface. The BG will be adsorbed on the catalyst surface and the high temperature can break the bonds of BG, e.g., C-H or C-C. At the same time, microwave irradiation can activate SZFO to be a semiconductor and generate electrons and holes which can oxidize BG to CO_2_ and H_2_O. Haneishi et al. (2019) researched the catalytic dehydrogenation reaction of 2-propanol into acetone using microwave (2.45 GHz) and conventional heating, respectively. It was found that the yield of acetone by MW irradiation were 19 and 12 times higher than that under conventional heating in 2.5 and 20 s. The reaction rate of microwave heating is also more than 10 times relative to conventional heating. The magnetite solid catalyst can absorb MW energy and generate a local high-temperature regions at the contact points which facilitates the reaction.

Transesterification is a kind of important chemical reaction which generally uses acid as catalyst. The liquid acid is poorly in catalytic performance and corrodes the equipment. A new microwave-assisted transesterification (MAT) technology with solid acid catalysts is used for transesterification. Nayak et al. (2019) reviewed the biodiesel synthesis by using microwave-assisted transesterification technology with various homogeneous and heterogeneous catalysts. Heterogeneous catalysts are easier to separate from the product and reuse compared to homogeneous catalysts. There are many types of heterogeneous catalysts, including metal oxides, mixed oxide, exchange membrane, carbon/ clay supported catalyst and enzymes mentioned below rather than homogeneous acid or base. The multiple selectivity of heterogeneous catalysts allows us to find the best catalyst in the appropriate reaction. H_2_SO_4_/C solid acid catalyst is selected for transesterification of biodiesel under microwave field (2.45 GHz, 200 W) (Yuan et al., 2009). Both activated carbon and sulfuric acid are of high dielectric properties and absorb microwave. H_2_SO_4_/C solid acid catalyst can also produce “microwave hot spots” under microwave irradiation, which means the temperature of catalyst is higher than that of liquid. In the case of heterogeneous acid catalysts, the high reaction temperature from “microwave hot spots” offsets the reduction of activity due to mass transfer resistance. In addition, the heterogeneous solid catalysts are able to react on the internal and external surfaces simultaneously. The high temperature is to the benefit of the endothermal reaction, so the yield of transesterification of biodiesel using H_2_SO_4_/C solid acid catalyst under microwave irradiation is improved. The results showed that microwave-assisted catalytic transesterification can reach a maximum yield of 94% compared with conventional heating. Prakash et al. (2015) compared the performance of glycerol and char (carbon particles) in the process of microwave pyrolysis. Its rated output is 800 W with a microwave frequency of 2,450 MHz. The results of experiments were that the yield obtained by using char as the microwave absorber is more than glycerol. The reason for this phenomenon is that char particles have a higher dielectric property than glycerol so that it can absorb more microwave energy. Therefore, char particles can act as energy sources to sustain the pyrolysis process and advance the reactions further.

The occurrence of solid phase reactions usually requires high temperature (Ren et al., 2015). Microwave irradiation can achieve high temperature conditions in a short time without introducing impurities. Pure ZnO and ZnO concentrate can't absorb microwave while carbon, as the reducing agent, can absorb microwave energy (Saidi and Azari, 2005). Microwave heating was carried out at a frequency of 2.45 GHz in two power levels, 900 and 1,000 W. The microwave energy absorbed by carbon can convert to heat which provides a high temperature environment for ZnO or ZnO concentrate. The electric loss factor of ZnO increases with the enhancement of surrounding temperature. The reduction reaction takes place in the critical temperature. The rate is increased and the product is reduced better under microwave irradiation.

The catalytic oxidation of toluene vapor by nano-size Co_3_O_4_ is conducted in the microwave field (Yi et al., 2018). The results showed that microwave irradiation can promote the removal rate of toluene in the low temperature. The interpretation for this phenomenon is the hypothesis of “hot spots.” Microwave irradiates heterogeneous nano-Co_3_O_4_ and increase the temperature of bulk surroundings. But the temperature of a certain micro-region is higher than the macroscopic temperature owing to the quick absorption of microwave energy. The solid catalyst provides a position for the conversion of active oxygen and oxygen vacancies. The special thermal effect makes the catalytic oxidation reaction continuous and efficient. Julian et al. (2019b) developed Mo-ZSM5 catalyst coated on silicon carbide monolith for methane under non-oxidative conditions. The resonant frequency of microwave is around 2.45 GHz. It can be observed that the temperature of gas is lower than that of catalyst under microwave heating in relative to the uniform temperature under homogeneous heating. The gas-solid temperature gradient can increase the selectivity of C_2_ and benzene and reduce the coke formation. They overcome the difficulties in microwave-assisted heterogeneous catalytic processes affected by coking and evaluated the scale of this process. It was found that a 6-fold increase in power can handle more than 150-fold reaction streams (Julian et al., 2019a). Table 1 presents the summary of the experimental content of the microwave local thermal effects in this chapter.

**Table 1:** Summary of special thermal effect of microwave on solid catalysts.

**Feed stock**	**Catalyst**	**Time/min**	**Temp/°C**	**MW/W**	**MW/MHz**	**Yield**
1-octanol	Magtrieve	25	360	–	–	99
Brilliant Green	spinel zinc ferrite	5	>1,000	360	2,450	99
2-propanol	magnetite catalyst	20	250	–	2,450	25
Fatty acid methyl ester	H_2_SO_4_/C	60	65	200	2,450	94
Bagasse	char	30	110	800	2,450	55
ZnO or ZnO concentrate	carbon	9	>1,000	1,000	2,450	>80
Toluene	Co_3_O_4_ nanoparticles	120	210	–	–	100
Methane	Mo-ZSM5	200	700	110	2,450	>40

## The Microwave Plasma

**Scheme 2:**
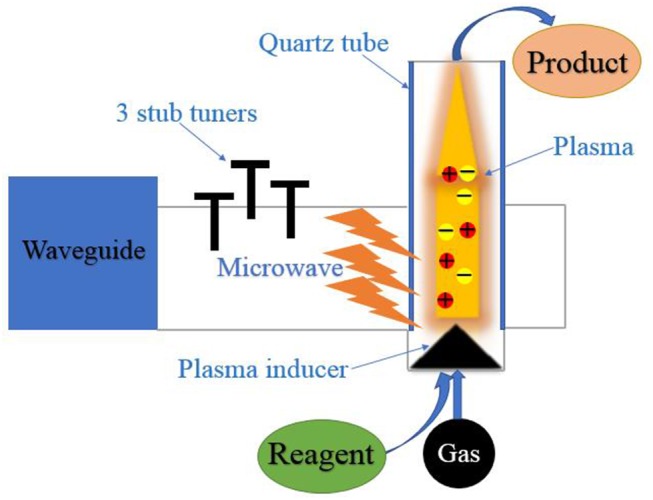
The schematic diagram of the microwave-induced plasma catalysis.

Microwave-induced plasmas (MIPs) technique has attracted wide attention in recent years (Moreno et al., 2019). The schematic diagram of microwave plasma generation is shown in Scheme 2. This technology has no requirement on the dielectric properties of solid phase, because the essence is partially ionized gas and energy storage (Douthwaite, 2007).

Microwave break down metal–dielectric powder mixture (Ti: CuO, etc.) and produce plasma (Batanov et al., 2018). The result showed that the ratio of metal and dielectric powder is important for discharge and chemical reaction. Pulsed microwaves can only sustain plasma with 1.3 ms in maximum. Then a solid-phase chemical reaction can initiate and be self-sustained by the plasma with high gas temperature. This process takes less time and initiate solid-phase reaction without solvent, while the microwave plasma-chemical technique can also be carried out in solvent. The antenna was heated by microwave (2.45 GHz, 500 W) to make the hydrocarbon vaporize and discharge. The gas bubbles with discharge will contact the suspending carbon powders which are good at absorbing microwave energy. Then the discharge phenomenon is disappeared. Through the analysis of composition and structure of solid and liquid phase, it is found that new nano-sized carbonaceous particles and polycyclic aromatic hydrocarbons are produced. The experiment results showed the microwave plasma can accelerate solid-phase reaction and synthesize high-quality products (Lebedev et al., 2014).

In additional, carbothermal method was used to synthesize molybdenum carbides (Mo_2_C). But the complex molybdenum salt precursor and excessive carbon will affect the quality of products. MPCVD (microwave-plasma chemical vapor deposition) process can overcome these drawbacks. Zhao et al. (2018) synthesized Mo_2_C by means of microwave plasma treated with methane and hydrogen mixed gases. The hydrogen plasma was excited by 920-W microwave irradiation with frequencies of 2.45 GHz between the two substrate holders. It will form an ellipsoidal plasma ball and can be controlled to heat the Mo foil on the substrates. The carbon atoms and molybdenum atoms in the vapor can generate Mo_2_C on the surface of Mo foil. The high temperature of reaction conditions can be provided by plasma self-heating. Under the condition of high temperature, the ionization of gases of plasma and the energy released by recombining free electrons and positive ions, which can meet the need of plasma glow discharge and stable temperature field. Meantime, the hydrogen plasma can etch the extra carbon on the surface of obtained samples to achieve pure products.

The application of microwave plasma in gas-phase is in the conversion of methane. The output power in the experiment is 300 W with a frequency of 2.45 GHz. Pt/Al_2_O_3_ is used as plasma catalyst to catalyze methane conversion (Nagazon and Yamaguchi, 2006). The result showed that the yields of ethylene and ethane have an increase on the condition of microwave irradiation. Pd–NiO/ɤ-Al_2_O_3_ and Pt–Sn/ɤ-Al_2_O_3_ are selected as plasma catalysts for the conversion reaction of natural gas to C_2_ products operating at a frequency of 2.45 GHz and a power of 1.2 kW (Cho et al., 2010). The mechanism of conversion is free radicals which are produced by electron–methane collisions. The addition of plasma catalyst can lead to an increase of the yield of C_2_ products from 47 to 63.7%. The hydrogasification of carbon at the presence of microwave plasma was further studied by Kim et al. (2007). They compared plasma reactions with Ar/H_2_ mixtures under different injection conditions. One is gas stream contacts with carbon outside the plasma area (post-plasma process), and another is the reaction of carbon particles and gas mixtures occur in the plasma area (plasma process). The plasma process was used a forward microwave power of 600 W and the post-plasma process was under power of 515 W at a fixed output frequency of 2.45 GHz. The experimental results showed that the production of plasma process is methane, acetylene and ethylene relative to the only product of methane from post-plasma process. The mechanism they concluded is the action of plasma energy and carbon particles can generate more C_2_ rather than H. Therefore, the most advantageous way for pure CH_4_ production is the post-plasma process. Chen et al. (2017) overviewed the CO_2_ conversion in a microwave discharge (915 MHz) for concluding the role of plasma catalysis. The combination of the microwave plasma and Ar plasma-treated NiO/TiO_2_ catalyst can significantly enhance the conversion and energy efficiency. The Ar plasma-treated NiO/TiO_2_ catalyst could result in a higher concentration of oxygen vacancies. And microwave plasma will facilitate the stepwise vibrational excitation of the CO_2_ molecule. The synergy leads to the improvement of reaction efficiencies. Table 2 presents the summary of the experimental content of the microwave plasma in this chapter.

**Table 2:** Summary of the microwave plasma.

**Feed stock**	**Time**	**Temp**	**MW (power)**	**MW (frequency)**
Ti: CuO	1.3 ms	1727–2727°C	40–50 kW	2.45 GHz
n-heptane	2 min	1427°C	500 W	2.45 GHz
Mo and carbon	–	–	920 W	2.45 GHz
Methane	–	27–327°C	300 W	2.45 GHz
Natural gas	40s	400–650°C	1,200 W	2.45 GHz
Hydrogasification of carbon	1 min	–	515 and 600 W	2.45 GHz
CO_2_	40 min	1327°C	–	915 MHz

## Enhanced Activity Site of Catalyst

The interfacial activity site of catalysts is a very important factor for the heterogeneous catalytic reaction. The activity site of strong acid ion-exchange resin could be enhanced by the microwave for the reaction from fructose into 5-HMF in acetone-water mixtures (Qi et al., 2008). A comparison was made between microwave and conventional heating, which illustrated that microwave field has a positive effect on the yield and selectivity of the product. The result showed that the yield of 5-HMF is more than 73.4% and the conversion of fructose is about 94% under microwave irradiation. They considered that the special effect of microwave and solid catalysts lead to this consequence. The strong acid ion-exchange resin consists of polar sulfo-group. The polar reaction mechanism is that the polar species of reactants will rotate fast in the microwave field and then molecules go from the ground state to the transition state more easily, which results in an increase of reactivity. Guo et al. (2012) compared six solid acid catalysts synthesized by carbonization and sulfonation for the conversion of fructose and glucose into 5-HMF. The lignin-derived solid acid catalyst showed the best catalytic performance under microwave irradiation owing to its high –SO_3_H group density and carbonization degree. The reaction is irradiated under microwave field of 100 W with temperature controlled at 100–160°C. In the presence of microwave field, the dipole–dipole type interaction between the dipolar solvent and polar reactants can produce electrostatic polar effects. In addition, microwave irradiation can strengthen electron transfer of sulphonic groups (Ji et al., 2018). As a result, the heat production and catalytic properties can be synergeticly enhanced.

**Scheme 3:**
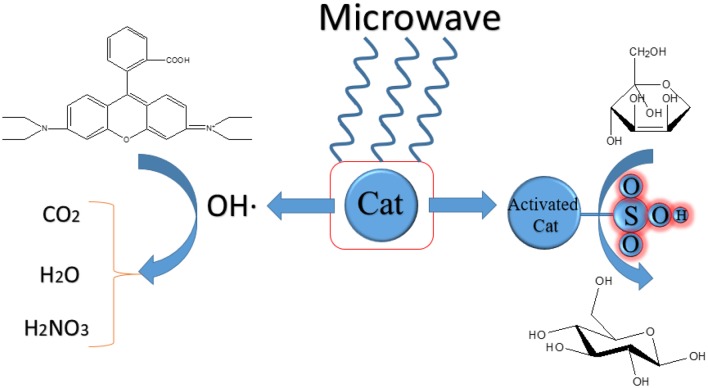
The schematic diagram of microwave on the catalyst activity.

In addition, there are still some unclear mechanisms in the reactions. CFA (coal fly ash) contains various metal oxides such as Fe, Cu, Mn, etc. which makes it can be Fenton-like catalyst and MW absorber. It can catalyze Fenton-like process to decolorize Rhodamine B (RhB) wastewater under the irradiation of microwave (Wang et al., 2019). The reason of decolorization is radicals and ·*OH* is more important than *HO*_2_· and ${O_{2}}^{-}$. CFA catalyzed Fenton-like process in the microwave field can achieve a higher degradation rate in a short time relative to other processes. The optimal MW power is 0.1 kW and the decolorization rate of RhB is more than 91%. The hot spot and possible non-thermal effect generated by microwave irradiation may be the interpretation of the increase of·*OH*. The exact mechanism of this reaction is remains to be verified. Table 3 presents the summary of the experimental content in this chapter. The mechanism of the effect of microwave on the catalyst activity is presumed as shown in the Scheme 3.

**Table 3:** Summary of enhanced activity site of microwave on catalyst.

**Feed stock**	**Time**	**Temp**	**MW (power)**	**Yield**
Fructose	20 min	100–180°C	–	73.4
Fructose and glucose	10 min	100–160°C	100 W	84
CFA	20 min	53°C	100 W	91.6

## The Microwave Enzyme Catalysis

Enzyme is a substance with high catalytic efficiency. The conditions of the enzymatic reaction are usually mild and can be carried out at low temperatures. The complex spatial structure of enzyme will affect its catalytic activity. At present, most of the explanations for microwave enzymatic reactions are from the perspective of structural changes. Microwave irradiation doesn't damage the nature of the enzyme and destroy the active site. Microwave will change the secondary structure and increase the flexibility of enzyme, which leads to a higher reaction rate. Mazinani et al. (2015) applied microwave-assisted method for trypsin-catalyzed hydrolysis. To verify the effect of microwave on enzyme, they chose azocasein, casein, and BAPNA [Na(±)-benzoyl-D/L-arginine-4-nitroanilide hydrochloride] for the digestion experiments by trypsin. This microwave reactor operates at a frequency of 2.45 GHz. The hydrolysis results were the same that microwave irradiation can increase the rate of reaction while the bulk temperature was kept at 25°C. The authors raised the possibility that the secondary structure of trypsin changes when exposed to microwave. Zhang et al. (2012) researched the hydrolysis process of starch by glucoamylase with 800 W microwave power at 2,450 MHz microwave radiation. By contrasting the SDS-PAGE (Sodium dodecyl sulfate-polyacrylamide gel electrophoresis) of microwave irradiation and water bath, the molecular weight of glucoamylase is the same which states peptide bonds of glucoamylase is not broken and microwave does not damage the primary structure of glucoamylase. But the circular dichroism (CD) spectra showed the peak height and peak position of microwave-treated samples have varying degrees of change compared to those of the untreated. Therefore, microwave irradiation can affect the hydrogen bonding and increase flexibility of glucoamylase, which may be the explanation of microwave promoting starch hydrolysis.

**Scheme 4:**
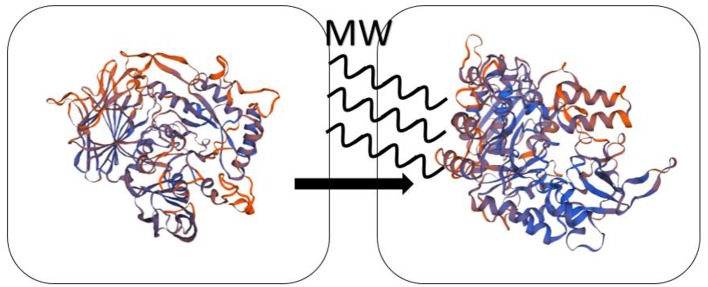
The schematic diagram of the microwave on the enzyme catalysis.

For the effect of microwave on enzyme, some scholars also gave their interpretations. Wang et al. (2018) catalyzed the synthesis of IOP (isooctyl palmitate) by immobilized lipase QLM under microwave irradiation. The microwave temperature and power were 70°C and 640 W, respectively. It showed that enzyme activity of immobilized QLM by microwave-assisted enzymatic reaction is about 9.6 times than that of free lipase QLM in conventional heating. Under optimum conditions of microwave irradiation, the yield of reaction is about 99% and the reaction time is only 3 h. The reason they speculated is that the polar domains of enzyme can change its flexibility after absorbing microwave energy in the microwave field, which dramatically increase the enzyme activity. Khambhala et al. (2018) treated inoculum for 2, 4, 6 min by microwave (90 W; 2,450 MHz) and compared with untreated sample. Microwave irradiation can change cellulase activity without genetically stability. The results showed that microwave-induced cellulase will lead the reversible nature of mutation owing to the non-thermal effect and micro-thermal effect. Díaz Ortiz et al. (2018) summarized the non-thermal effect of microwave in photochemical reaction is that microwave can accelerate photoinduction electron transfer. The computational calculations showed that the polarizability of transition states and the stabilization of radicals and triplet states can be influenced through non-thermal effects. Micro-thermal effect, proposed by Shamis et al. (2012), is different from the thermal effect of bulk temperature changes. They found that the activity of both LDH (lactate dehydrogenase) and COX (cytochrome coxidase) increases under microwave radiation while the bulk temperature is constant. Table 4 presents the summary of the experimental content of the microwave enzyme catalysis in this chapter. The mechanism of the effect of microwave on the enzyme catalysis is presumed as shown in Scheme 4.

**Table 4:** Summary of microwave enzyme catalysis.

**Feed stock**	**enzyme**	**Time/min**	**Temp/°C**	**MW/W**	**MW/MHz**	**Yield/%**
Casein	Trypsin	5	25	20	2,450	80
Starch	Glucoamylase	60	62	800	2,450	–
Palmitic acid, 2-ethyl hexanol	Thermophilic lipase QLM	240	70	640	–	99
Brevibacillus parabrevis	Cellulase	30–60	50–121	90	2,450	87.5

## Concluding Remarks

It is well-known that microwave irradiation can produce several special effects for reaction intensification owing to dielectric heating. However, the effect of microwave on substances for enhanced reaction is still under discussion. In this context, there are some different mechanisms of microwave and heterogeneous catalysts. The interaction between microwave and solid catalysts can produce local thermal effect as well as special effect. Microwave-induced plasmas (MIPs) technique is considered as an effective way to promote the solid/gas phase reaction. Enzymatic reactions under microwave irradiation can usually be attributed to the change of structure of enzyme. The results of these experiment show that microwave can effectively promote the reaction and increase the yield. This review can provide a method of microwave irradiation to facilitate the heterogeneous catalytic reactions. Furthermore, it summarizes the current mechanism of microwave with different forms of substances, which can give some explanatory ideas for the following scholars.

However, current microwave-assisted catalytic reactions stay on a lab-scale instead of large-scale production. There are some limitations for the industrial applications of microwave field. Gutiérrez-Acebo et al. (2018) researched the effect of microwave heating and conventional heating on sulfonic acid-functionalizing solid catalysts. Then the catalysts are used for the acetalization of glycerol with furfural. It is surprising observed that catalysts prepared with microwaves led to higher conversion but slight lower selectivity compared with the catalyst prepared by conventional heating. The results illustrated that the microwave will affect the properties of catalysts during sulfonation. According to the mechanism of reaction transformation, it is inferred that microwave will reduce Brønsted acid and Lewis acid sites. This reveals that microwaves are not beneficial in all respects. Stankiewicz et al. (2018) discussed the possibility of applying microwave in industry. Large-scale production needs increase dimensions and volume of reaction equipment. Owing to the extensive scale-up, the frequency of microwave must shift from 2.45 GHz to 915 MHz. The shift of frequency requires massive studies about physical transport, frequency-related microwave-catalyst interaction, and reaction performance. In addition, the microwave penetration depth and energy distribution are also to be solved. Priecel and Lopez-Sanchez (2018) also doubt the reality for scaling up of microwave assisted reactions. Microwave system can't simply incorporate into the current engineering owing to the significant investment, which is a big limiting factor. It is beneficial to shift the frequency to 915 MHz because extensive reactor construction needs satisfy penetration depth. Even the type and arrangement of reactors are required to change accordingly. Moreover, reaction time, energy efficiency and temperature measurement are also issues that are expected to be solved.

Therefore, there is no exact data to prove the existing conjecture and some interpretations are still controversial. In the future, we expect that more applications of microwave in other fields and more scholars can discuss the mechanism of microwave to better meet the needs of heterogeneous catalytic reactions. The large-scale industrial applications of microwaves also require more basic research support.

## Author Contributions

HL and XG designed the review idea. CZ, CP, and XG collected literature. CZ and XG wrote the paper. XG and XL revised this paper.

## Conflict of Interest

The authors declare that the research was conducted in the absence of any commercial or financial relationships that could be construed as a potential conflict of interest.
